# Anti-inflammatory role of fenofibrate in treating diseases

**DOI:** 10.17305/bb.2022.8534

**Published:** 2023-05-01

**Authors:** Lv Jin, Hu Hua, Yong Ji, Zhanjun Jia, Mingqi Peng, Songming Huang

**Affiliations:** 1 Department of Nephrology, Children’s Hospital of Nanjing Medical University, Nanjing, China; 2 Nanjing Key Laboratory of Pediatrics, Children’s Hospital of Nanjing Medical University, Nanjing, China; 3 Jiangsu Key Laboratory of Pediatrics, Nanjing Medical University, Nanjing, China; 4 Nursing Department, Children’s Hospital of Nanjing Medical University, Nanjing, China

**Keywords:** Fenofibrate, inflammation, mechanism, peroxisome proliferator-activated receptor-α (PPAR-α), diseases, therapy

## Abstract

Inflammation contributes to the pathogenesis of several diseases. Fenofibrate, known as a peroxisome proliferator-activated receptor-α (PPAR-α) agonist, is a classic drug for treating hyperlipidemia. In addition to its lipid-lowering effect, fenofibrate has also been reported to exert anti-inflammatory effects with complicated underlying mechanisms of action. In general, the anti-inflammatory effect of fenofibrate is secondary to its lipid-lowering effect, especially for the inflammation caused by hyperlipidemia in the circulatory system. Some anti-inflammatory actions may also come from its regulatory effects on intracellular lipid metabolism by activating PPAR-α. In addition, some roles in anti-inflammation might be mediated by its direct regulation of inflammatory signaling pathways. In order to understand anti-inflammatory activities and the underlying mechanisms of fenofibrate action in disease better, we herein reviewed and discussed the anti-inflammatory roles and its subserving mechanisms in various diseases of different organ systems. Thus, this review offers insights into the optimal use of fenofibrate in the clinical setting.

## Introduction

Fenofibrate is a peroxisome proliferator-activated receptor-α (PPAR-α) agonist approved by the Food and Drug Administration (FDA) to routinely treat hyperlipidemia with elevated triglycerides (TGs), or mixed hyperlipidemia with elevated TGs and reduced high-density lipoprotein cholesterol (HDL-C) levels [1]. In addition to lowering blood lipids, fenofibrate can significantly reduce the risk of major cardiovascular events [2]. Researchers have demonstrated that fenofibrate exerts protective effects against diabetes and diabetes-associated pathologies [3]. Fenofibrate safeguards epithelial function and reduces vascular complications, including diabetic retinopathy (DR), nephropathy, and cardiopathy [4–6], in addition to being used to rescue diabetes-related organ impairment [7]. Fenofibrate also inhibits the development of liver diseases, including non-alcoholic fatty liver disease (NAFLD), steatohepatitis, hepatitis C virus (HCV), and cholestatic hepatitis [8–11]. Recent evidence shows that fenofibrate can suppress many types of human cancers [12], and even drug-induced disorders [13, 14]. All these therapeutic effects of fenofibrate might arise from its anti-inflammatory properties.

Although inflammation is a defensive response of the body to stimulation, continued or excessive inflammation may result in adverse events [15]. Inflammation has been demonstrated to promote the progression of several diseases, such as obesity, diabetes [16], cancer [17], cardiovascular disease [18], eye disorders [19], arthritis [20], neuroinflammation [21], autoimmune diseases [22], and inflammatory bowel disease [23]. Furthermore, several studies have revealed that fenofibrate exhibits a robust anti-inflammatory effect on diseases of various systems. This work presents the latest research on anti-inflammatory and other similar effects of fenofibrate.

## Indirect and direct anti-inflammatory effects of fenofibrate

The anti-inflammatory effects and mechanisms of fenofibrate vary across disease types. Roughly, its anti-inflammatory effects can be divided into indirect and direct. Lipid metabolism is disrupted in an inflammatory environment in vivo [24], and fenofibrate may repress inflammation to maintain a normal metabolic status. Fenofibrate upregulates the expression of HDL and downregulates that of low-density lipoprotein (LDL) [1]. The expression of HDL apolipoprotein A-I (ApoA-I) can be upregulated by fenofibrate to inhibit atherosclerotic progression [25]. By reducing cholesterol accumulation in the liver, fenofibrate attenuates hepatic inflammation and inhibits the progression of non-alcoholic steatohepatitis (NASH) [26]. Very-long-chain sphingolipid can also be raised by fenofibrate to relieve inflammation [3, 27]. These studies prove that fenofibrate can protect against inflammation in an indirect manner.

Fenofibrate also has a direct anti-inflammatory effect. Independent of its lipid-lowering effects, fenofibrate counters inflammation primarily by activating PPAR-α which plays a key role in both lipid metabolism and inflammation. Upon PPAR-α activation, fenofibrate suppresses inflammatory pathways involving nuclear factor kappa-B (NF-κB) [28], sirtuin 1 (SIRT1) [29], toll-like receptor 4 (TLR4) [30], adenosine mitogen-activated protein kinase (AMPK) [31], or interleukin IL-1 or IL-6 [32]. Fenofibrate also directly inhibits the expression of inflammation-related genes in inflammatory pathways. In addition, the attenuation of mitochondrial disfunction [33] and the differentiation of T helper cells 17 (Th17) also assist in combating inflammation [34]. In summary, fenofibrate exerts its anti-inflammatory effects either directly or indirectly.

## Anti-inflammatory mechanism of fenofibrate

Fenofibrate has been reported to have anti-inflammatory effects in several diseases. In this part, the mechanism of how fenofibrate works against inflammation will be introduced in various diseases of different organ systems.

### Endocrine system diseases

#### Obesity

Fenofibrate can attenuate the inflammation involved in obesity. A positive correlation between inflammation or reactive oxygen species (ROS) production and peroxisomal dysfunction has been observed in mice with high-fat diet (HFD)-induced obesity. Relying on PPAR-α to repair peroxisomal function, fenofibrate inhibited inflammation in these obese mice [35]. Moreover, in 3T3-L1 adipocytes, fenofibrate increased SIRT1 expression and suppressed the NF-κB pathway by activating PPAR-α and boosting AMPK phosphorylation. Subsequently, fenofibrate-inhibited tumor necrosis factor-α (TNF-α)-induced CD40 expression [29]. Fenofibrate-upregulated SIRT1 expression is associated with a diminution in markers of inflammation, including high-sensitivity C reactive protein (hs-CRP), IL-6, and fetuin-A, in obese patients with or without type 2 diabetes [36]. In addition, fenofibrate increased the expression of adiponectin, which was secreted by adipocytes and able to mediate the interrelationship among adiposity, insulin resistance, and inflammation [37, 38]. At present, fenofibrate is not the main drug to treat obesity, but in some obese patients with hyperlipidemia, fenofibrate may achieve evident effects.

In general, fenofibrate can inhibit the inflammation in obesity, a mechanism that often involves the activation of PPAR-α.

#### Diabetes

In diabetes, the abnormal glucose metabolism may cause systematic inflammation. In patients with pre-diabetes, the plasma markers of inflammation and monocytic secretory function increase significantly [39]. Fenofibrate shows favorable effects in reducing the cytokine-provoked release of TNF-α, IL-1, IL-6, monocyte chemoattractant protein 1 (MCP-1), and plasma hs-CRP from monocytes in patients with pre-diabetes [39, 40]. Further, the anti-inflammatory effects of fenofibrate improve diabetic patients’ sensitivity to insulin [39]. In patients with metabolic syndrome, the reduction of systemic inflammation markers by fenofibrate is more dependent on the activation of PPAR—than on the regulation of lipid and glucose metabolism and insulin sensitivity [41].

Fenofibrate enhances anti-inflammatory processes in type 1 and type 2 diabetes patients [27, 42]. In type 2 diabetes, β-cell function is damaged by inflammation. A recent study has revealed that fenofibrate ameliorates lipotoxicity-induced β-cell disfunction and apoptosis in lipoprotein lipase (LPL)+/– mice and palmitate (PA)-treated stable mouse insulinoma 6 (MIN6) cells, which is achieved by the inhibition of NF-κB/MIF (macrophage-inhibitory factor) inflammatory pathway [28]. Abnormal metabolism of sphingolipids in type 1 diabetes can also lead to β-cell dysfunction. Fenofibrate upregulates the expression of very-long-chain sphingolipids in non-obese diabetic (NOD) mice and remodels pancreatic lipidome into a more anti-inflammatory state [3, 27].

In type 2 diabetes patients with hypertriglyceridemia, fenofibrate decreased the levels of reduced upon activation, normal T cell expressed and secreted (RANTES) and inhibited inflammatory responses [43]. In addition, both fenofibrate and its phase-I bio-transformed metabolite fenofibric acid weakened COX-2 enzymatic activity, thus generating anti-inflammatory effects both in vivo and in vitro. Moreover, fenofibric acid exhibited potent time-dependent anti-inflammatory effects [44].

Interestingly, fenofibrate reduced systematic inflammation in diabetes by protecting β-cell functions. The inhibition of the COX-2 enzyme and the decrease in RANTES cooperated to repress inflammatory responses. Whether insulin sensitivity is linked to the anti-inflammatory effects of fenofibrate remains to be verified by further studies. At present, fenofibrate is not routinely used to treat diabetes in clinic. Whether it can be combined with other drugs to treat diabetes or hyperlipidemic diabetes patients deserves more in-depth research.

In summary, fenofibrate can significantly improve diabetes-related inflammation through different mechanisms.

#### Hyperlipidemia

Abnormal blood lipid metabolism may lead to hyperlipidemia. Hyperlipidemia induces an elevation in inflammatory factors and subsequently a chronic inflammation state. Fenofibrate can reduce the plasma concentrations of inflammatory markers, including (hs-CRP) and fibrinogen, in hyperlipidemic patients [45]. This reduction may involve the upregulation of adiponectin by fenofibrate [46]. In hypercholesterolemic rabbits, fenofibrate lowers the levels of plasma acute-phase proteins, a process associated with the reduction in TNF-α in adipocytes [47]. In addition, fenofibrate regulates DNA methylation to control inflammatory response. Downregulation of hs-CRP, IL-2, and IL-6 is associated with multiple cytosine-guanine (CpG) sites [48].

Fenofibrate improved both HFD-induced insulin resistance in skeletal muscle and palmitic acid-induced insulin resistance in myotube cells, thus reduced ER stress-induced inflammation via inhibiting TLR4/NF-κB pathway [30]. Moreover, by activating PPAR-α, fenofibrate suppresses the NF-κB P65 pathway and renders anti-inflammatory effects in patients with acute hypertriglyceridemic pancreatitis [49]. Fenofibrate is commonly used for hyperlipidemia in clinic.

In summary, fenofibrate can significantly curb hyperlipidemia-related inflammation. This effect is partially linked to fenofibrate-induced reduction of lipids in the serum (Table 1).

**Table 1:** Anti-inflammatory targets and mechanisms of fenofibrate in endocrine system diseases

**Diseases**	**Cells/Models**	**Targets and mechanisms**	**Implications**	**References**
Obesity	Mouse	PPAR-α↑-peroxisomal dysfunction*↓*	Reduce inflammation	[35]
	3T3-L1 adipocytes	PPAR-α↑-AMPK phosphorylation*↑*-SIRT1*↑*-NF-κB*↓*-TNF-α-induced CD40*↓*	Reduce proinflammatory factor CD40	[29]
	Human	SIRT1*↑*	Regulate markers of inflammation	[36]
	Mouse, 3T3-L1 adipocytes	Adiponectin*↑*	Reduce inflammation	[37, 38]
Diabetes	Human	Insulin sensitivity*↑*	Have early anti-inflammatory effect	[39]
	Human	PPAR-α↑	Reduce inflammatory responses	[41]
	LPL+/– mouse and PA-treated MIN6 cells	NF-κB/MIF inflammatory pathway*↓*	Ameliorate lipotoxicity-induced β-cell dysfunction and apoptosis	[28]
	NOD mouse	Very-long-chain sphingolipid*↑*	Alter the pancreatic lipidome to a more anti-inflammatory state	[3, 27]
	Human	RANTES*↓*	Restrain the inflammatory responses	[43]
	Rat	COX-2 enzyme*↓*	Have anti-inflammatory effects both in vivo and in vitro	[44]
Dyslipidemia	Human	Adiponectin*↑*	Reduce inflammation	[46]
	Rabbit	TNF-α in adipocytes*↓*-Acute-phase proteins*↓*	Reduce inflammatory responses	[47]
	CD4+ T cells	DNA methylation	Reduce inflammatory responses	[48]
	Rat, Differentiated C2C12 myotubes	TLR4/NF-κB pathway*↓*	Reduce ER stress-induced inflammation	[30]
	Human	PPAR-α↑-NF-κB P65*↓*	Reduce inflammatory responses	[49]

PRAR-α: Peroxisome proliferator-activated receptors; AMPK: Adenosine monophosphate activated protein kinase; SIRT1: Sirtuin 1; NF-κB: Nuclear factor κB; TNF-α: Tumor necrosis factor α; MIF: Macrophage migration inhibitory factor; RANTES: Regulated upon activation, normal T cell expressed and secreted; COX-2: Cyclooxygenase-2; TLR4: Toll-like receptor-4; LPL: Lipoprotein lipase; NOD: Non-obese diabetic.

### Circulatory system diseases

#### Atherosclerosis

Inflammation is closely associated with atherosclerosis and its complications [50]. Fenofibrate has demonstrated the ability to effectively attenuate inflammation in atherosclerosis [51], which may be associated with its lipid-lowering effects [52].

Fenofibrate reduces the levels of CRP and the CRP-induced cytokines, including MCP-1 [53], as well as the expression of CD40 and CD40L [54]. The downregulation of the CD40–CD40L-signaling pathway inactivates matrix metalloproteinase 2 (MMP-2) and MMP-9 in human umbilical vein endothelial cells (HUVECs) and reduces inflammatory responses in atherosclerosis [54]. Fenofibrate weakens the proinflammatory effects of cytokines in vascular cells, thus preventing the progression of atherosclerosis [53]. In ApoE*3Leiden transgenic mice fed with high levels of cholesterol, investigators have discerned a reduction in the levels of proinflammatory chemokine MCP-1, intercellular cell adhesion molecule 1 (ICAM-1), and monocyte/macrophage differentiation factor granulocyte-macrophage colony-stimulating factor (GM-CSF) after fenofibrate treatment, indicating that fenofibrate can inhibit the adhesion, recruitment, and maturation of monocytes/macrophages and the inflammation of vascular components. The inhibition on p65-NF-κB signaling is possibly associated with a reduction in monocyte adhesion induced by fenofibrate [55].

Fenofibrate can activate PPAR-α to inhibit atherosclerotic progression. Inflammatory cytokines induce an increase in serum amyloid (SAA) and a decrease in ApoA-I and paraoxonase 1 (PON-1) mRNA expression by suppressing PPAR-α activity. These effects can be reversed by fenofibrate in a PPAR-α-dependent manner, thereby relieving atherosclerosis [25]. By activating PPAR-α, fenofibrate also induces heme oxygenase-1 (HO-1, an anti-inflammatory enzyme) expression in human vascular endothelial and smooth muscle cells [56]. Platelet-activating factor (PAF) is a proinflammatory molecule intimately involved in the progression of atherosclerosis [57]. Fenofibrate decreases the level of PAF receptors in human monocytes and macrophages in a PPAR-α-dependent fashion [58], a process mediated by β-defensin 1. The activation of PPAR-α upregulates the secretion of β-defensin 1 and suppresses TLR4. Through PPAR-α/β-defensin1/TLR4 pathways, fenofibrate inhibits the activation of macrophages induced by lipopolysaccharide (LPS) [59]. Fenofibrate also suppresses TLR4 during angiotensin (Ang) II-induced inflammation in vascular smooth muscle cells (VSMCs). In addition, fenofibrate reduces inflammatory responses of VSMCs by interfering with the TLR4-dependent-signaling pathway (TLR4/IP-10/PKC/NF-kB) [60]. Moreover, the inhibition of another interferon-β (TRIF)-dependent-signaling pathway (TLR4/TRIF/IRF3/IP-10) by fenofibrate was found to assist in reducing inflammatory responses in LPS-induced VSMCs [61]. PPAR-α activation induced by fenofibrate leads to the high expression of SIRT1 and SIRT1-mediated deacetylation of forkhead box O1 (FoxO1). PPAR-α, SIRT1, and FoxO1 co-work to inhibit cellular apoptosis induced by TNF-α in vascular adventitial fibroblasts (VAFs), indicating that fenofibrate can regulate atherosclerosis-associated inflammation to counterstrike cellular apoptosis [62].

The aforementioned results collectively reveal that fenofibrate can attenuate inflammation to block the progression of atherosclerosis. These anti-inflammatory effects are associated with its induction on PPAR-α and subsequent inhibition on inflammatory pathways, rather than fenofibrate’s lipid-lowering activities. However, whether fenofibrate can be used for clinical management of atherosclerosis-related inflammation needs human studies or clinical trials.

#### Abdominal aortic aneurysm

Abdominal aortic aneurysm (AAA) is a serious condition associated with aortic inflammation. Fenofibrate induces the activation of the sphingosine 1 phosphate (S1P) pathway and the nitric oxide (NO) pathway in mice with Ang II-induced AAA, thereby reducing aortic inflammation [63]. This mechanism also acts to inhibit the progression of atherosclerosis.

#### Diabetic retinopathy

DR is a microvascular disease associated with inflammation of blood vessels. Fenofibrate reduces the inflammation in DR via both PPAR-α-dependent and -independent mechanisms [31, 64].

The overexpression of PPAR-α in the retina mitigates retinal vascular leakage and retinal inflammation induced by diabetes [65]. By activating PPAR-α, fenofibrate induced direct effects on inhibiting proinflammatory MCP-1, ICAM-1, and transcription factor NF-kB [66]. Studies have revealed that the inhibition on NF-κB reduced the production of the inflammatory chemokines MCP-1, fractalkine (FKN), and ICAM-1, as well as the oxidative products in DR [67, 68]. Enright et al. [69] have demonstrated that fenofibrate activates PPAR-α in the liver, but not in the retina, to modulate circulating cytokines, growth factors, and/or lipids, suggesting that fenofibrate indirectly affects the retina by inducing the expression of genes related to the local inflammatory response.

Fenofibrate also directly activates AMPK in human glomerular microvascular endothelial cells (HGMECs), thus elevating NO production and reducing inflammation [31]. Angiopoietin-like 3 (ANGPTL3) and its accompanying inflammation were closely linked to the pathogenesis of DR [70]. Moreover, fenofibrate attenuates oxidative stress by upregulating nuclear factor erythroid-2-related factor 2 (Nrf2) signaling in Müller cells. Fenofibrate also downregulates the NOD-like receptor thermal protein domain associated protein 3 (NLRP3) inflammasome to reduced caspase-1 and pro-IL-1β, thus generating anti-inflammatory effects in DR. However, whether PPAR-α plays a role in this mechanism remains unknown [71]. In addition, adiponectin was demonstrated to mediate proinflammatory effects in DR. Fenofibrate generated significant anti-inflammatory effects and inhibited the progression of DR by reducing the expression of adiponectin and its receptors in DR [72]. Fenofibric acid, a metabolite of fenofibrate, inhibits the expression of COX-2 and inflammatory factors in DR [73]. At present, fenofibrate is not used for diabetes in clinic. In general, fenofibrate employs its anti-inflammatory effects to protect from DR.

#### Cardiac dysfunction

Inflammation is involved in cardiac disfunction induced by primary diseases. Suppressing inflammation slows down the progression of heart diseases, such as heart failure [74] and myocardial infarction [75].

In cardiac dysfunction induced by high glucose, fenofibrate represses inflammatory responses by upregulating fibroblast growth factor 21 (FGF21) and promoting SIRT1-mediated autophagy in heart tissues [76]. The function of high-mobility group box 1 (HMGB1) protein depends upon its location, and extracellular HMGB1 works as a delayed mediator of proinflammatory cytokines in the initiation and amplification of inflammatory responses, whereas nuclear HMGB1 prevents cardiac hypertrophy and heart failure. Indirectly, fenofibrate activates PPAR-α to modulate both the expression and location of HMGB1, thus arousing anti-inflammatory response against cardiac hypertrophy [77]. In addition, fenofibrate attenuates doxorubicin (DOX)-induced cardiac inflammation in mice by activating the endothelial NOS/EPC pathway [78] and quelling the NF-κB pathway [79]. Fenofibrate also stimulates PPAR-α to relieve myocardial inflammation induced by Ang II. The expression level of NF-kB is downregulated and those of vascular cell adhesion molecule-1 (VCAM-1), platelet endothelial cell adhesion molecule (PECAM), and ICAM-1 are upregulated after fenofibrate intervention [80]. Fenofibrate blunts myocarditis by increasing the expression of the anti-inflammatory cytokine IL-10 [81].

In patients with chronic heart failure (CHF), fenofibrate directly blocks the interaction between monocytes and human aortic endothelial cells (HAECs) as TNF-α is activated. Fenofibrate also reduces VCAM-1 and ICAM-1 expression. It appears that fenofibrate decreases the expression level of cell adhesion molecules to inhibit inflammation in vascular tissues of patients with CHF [82]. Furthermore, fenofibrate hinders the infiltration of macrophages and T lymphocytes into the left ventricle of the heart. These anti-inflammatory effects contribute to the prevention of heart failure [83]. These studies collectively show that fenofibrate counters inflammation to safeguard cardiac function; however, whether it can be applied to clinical practice needs further exploration (Table 2).

**Table 2:** Anti-inflammatory targets and mechanisms underlying fenofibrate action in circulatory system diseases

**Diseases**	**Cells/Models**	**Targets and mechanisms**	**Implications**	**References**
Atherosclerosis	HUVECs	MCP-1*↓*	Reduce the inflammation of atherosclerosis	[53]
	HUVECs	CD40-CD40L*↓*-MMP-2*↓*, MMP-9*↓*	Reduce the inflammation of atherosclerosis	[54]
	Mouse	p65-NF-κB*↓*	Reduce monocyte adhesion	[55]
	Murine hepatoma cells	PPAR-α↑- SAA*↓*, apoA-I and PON-1*↑*	Reverse the inflammatory cytokine effects	[25]
	Human vascular endothelial and smooth muscle cells	PPAR-α↑-HO-1*↑*	Reduce the inflammation of atherosclerosis	[56]
	Human monocytes and macrophages	PPAR-α↑, PAF receptor gene*↓*	Reduce proinflammatory molecule and inhibit progression of atherosclerosis	[58]
	The murine macrophage-like cell line J774	PPAR-α↑-β-defensin1*↑*-TLR4*↓*	Inhibit the inflammatory activation of macrophages	[59]
	Rat, VSMCs	TLR4/IP-10/PKC/NF-κB pathway*↓*	Reduce the inflammation of atherosclerosis	[60]
	VAMCs	TLR4/TRIF/IRF3/IP-10 pathway*↓*	Reduce the inflammation of atherosclerosis	[61]
	Mouse, rat VAFs	PPAR-α↑-SIRT1*↑*-deacetylation of FoxO1s*↑*	Reduce cellular apoptosis and regulate the inflammation associated with atherosclerosis	[62]
Abdominal aortic aneurysm	Mouse	S1P pathway*↑*-NO pathway*↑*	Reduce aortic inflammation	[63]
Diabetic retinopathy	Rat	PPAR-α↑	Mitigate retinal vascular leakage and retinal inflammation	[65]
	Rat, human retinal pigment epithelium cells	PPAR-α↑-NF-κB*↓*	Reduce the production of the inflammatory chemokines and inhibit oxidative products in DR	[67, 68]
	Mouse	PPAR-α in the liver*↑*	Modulate circulating cytokines, growth factors, and/or lipids	[69]
	HGMECs	AMPK*↑*	Elevate NO production and reduce inflammation	[31]
	Rat, HRMECs	ANGPTL3 pathway*↓*	Reduce its accompanying inflammation	[70]
	Mouse	Nrf2 expression*↑*, NLRP3 inflammasome activation*↓*	Attenuate oxidative stress, reduced caspase-1 and pro-IL-1β	[71]
	Rat, RAW264.7 cells, RGC-5 cells	Adiponectin and its receptors*↓*	Have anti-inflammatory effects	[72]
	RRECs	COX-2*↓*	Have anti-inflammatory effects	[73]
Cardiac dysfunction	Mouse	FGF21*↑*-SIRT1-mediated autophagy*↑*	Reduce cardiac inflammatory responses	[76]
	Rat cardiomyocytes, mouse	HMGB1*↓*, Extracellular HMGB1*↓*	Facilitate anti-inflammatory responses	[77]
	Mouse	eNOS/EPC pathway*↑*	Attenuate DOX-induced cardiac inflammation	[78]
	Mouse	NF-κB pathway*↓*	Attenuate DOX-induced cardiac inflammation	[79]
	Rat	PPAR-α↑	Have anti-inflammatory effects	[80]
	Rat	IL-10*↑*	Inhibit the progression of myocarditis	[81]
	PBMCs, HAECs	VCAM-1 and ICAM-1*↓*- monocyte binding HAECs	Attenuate the inflammation seen in vascular tissues	[82]
	Rat	Infiltration of macrophages and T lymphocytes*↓*	Abatement of heart failure progression	[83]

HUVEC: Human umbilical vein endothelial cell; MCP-1: Monocyte chemoattractant protein 1; MMP-2: Matrix metalloproteinase 2; MMP-9 Matrix metalloproteinase 9; NF-κB: Nuclear factor κ-B; PPAR-α: Peroxisome proliferator-activated receptor-α; SAA: Serum amyloid; ApoA-I: Apolipoprotein A-I; PON-1: Paraoxonase 1; HO-1: Heme oxygenase-1; PAF: Platelet-activating factor; TLR4: Toll-like receptor 4; VSMC: Vascular smooth muscle cell; SIRT1: Sirtuin 1; FoxO1: Forkhead box O1; S1P: Sphingosine 1 phosphate; NO: Nitric oxide; AMPK: Adenosine mitogen-activated protein kinase; HGMEC: Human glomerular microvascular endothelial cell; ANGPTL3: Angiopoietin-like 3; Nrf2: Nuclear factor erythroid-2-related factor 2; NLRP3: NOD-like receptor thermal protein domain associated protein 3; FGF21: Fibroblast growth factor 21; HMGB1: High-mobility group box 1; DOX: Doxorubicin; IL: Interleukin; VCAM-1: Vascular cell adhesion molecule-1; ICAM-1: Intercellular cell adhesion molecule 1; HAEC: Human aortic endothelial cell.

### Digestive system diseases

#### Liver diseases

The liver is susceptible to inflammation. Fenofibrate can treat inflammation in NASH, cholestatic liver injury, liver ischemia–reperfusion (I/R) injury, etc.

NASH is a chronic liver disease that is characterized by hepatic steatosis, lobular inflammation, hepatocyte ballooning, and fibrosis. The liver is the site where multiple metabolic pathways occur. Cholesterol is primarily metabolized in the liver and the accumulation of cholesterol in the liver could lead to NASH. Fenofibrate can reverse the downregulation of sterol-regulatory element binding proteins (SREBPs), liver X receptors (LXRs), and their target genes, thereafter contributing to cholesterol catabolism [84]. In this manner, fenofibrate can reduce the cholesterol accumulation in the liver and inhibit inflammation in NASH [26]. The downregulation of PPAR-α is a feature of NASH, and PPAR-α activation prevents the accumulation of lipid and the secretion of modulating inflammatory chemokines [85]. Fenofibrate activates PPAR-α and its target genes to prevent NASH in a murine model of 3,5-diethoxycarbonyl-1,4-dihydrocollidine (DDC)-induced steatohepatitis-like hepatocellular damage [86] and a mouse model of HFD-fed NASH [87]. In addition, fenofibrate exerted a direct anti-inflammation effect in NASH by regulating NF-kB and its target inflammatory genes [9]. These results suggest that fenofibrate induces anti-inflammatory effects through directly regulating lipid metabolism and inflammatory pathways.

Sedimentation of bile acids in the liver can lead to liver toxicity followed by inflammatory responses. Relying on PPAR-α, fenofibrate reduces the production of bile acids and keeps them nontoxic in patients with primary biliary cholangitis (PBC) or primary sclerosing cholangitis (PSC). The proinflammatory cytokines induced by bile acids are then reduced after fenofibrate treatment [88]. In rats with bile duct ligation (BDL), fenofibrate effectively downregulates serum cytokine to ameliorating hepatic inflammation. However, proliferation of the bile duct becomes more marked after fenofibrate treatment [89], but whether this effect leads to side effects in patients remains unknown. In addition, fenofibrate prevents cholestatic liver injury induced by α-naphthyl isothiocyanate (ANIT) by upregulating the expression of genes associated with fatty acid β-oxidation (β-FAO), including carnitine palmitoyltransferase 1b (Cpt1b), carnitine palmitoyltransferase 2 (Cpt2), and medium-chain acyl-CoA dehydrogenase (Mcad) [90]. The anti-inflammatory effects of fenofibrate also demonstrated to recover liver dysfunction in liver I/R injury. Besides, it restores serum levels of alanine aminotransferase (ALT) and TNF-α and suppresses oxidative stress, necrosis, and apoptosis in ischemic liver tissues [91].

Fenofibrate shows evident efficacy in treating multiple typical inflammatory hepatocellular adenomas. Given that inflammatory hepatocellular adenoma is characterized by the activation of the IL-6/JAK/STAT pathway, we suspect that this efficacy may be associated with the suppression of IL-6 [92].

Adiponectin receptors are closely linked with hepatic inflammation. In a model of HCV-induced steatosis, fenofibrate rescues the expression of pAMPK and adiponectin receptor 2 (AdipoR2) by reducing ER stress and inflammatory proteins [93]. In addition to liver inflammation, fenofibrate was reported to reduce systemic inflammation in the systemic acute-phase response in mice by activating PPAR-α and reducing the expression of IL-6 [32, 94]. In conclusion, fenofibrate can significantly relieve liver disease-related inflammation, but its clinical application lacks sufficient evidence.

#### Intestinal inflammation

Enteritis and colitis, usually caused by bacteria, viruses, fungi, and parasites, involve the abnormal Th17 activities in the intestines. Fenofibrate suppresses the differentiation of Th17 cells independent of PPAR-α [95]. In T and B lymphocytes and colonic epithelial cells, fenofibrate decreases the secretion of interferon-γ (IFN-γ) and IL-17 from Th1 and Th17 cells in colitis by activating PPAR-α. Moreover, fenofibrate represses the expression of chemokines C-X-C motif chemokine ligand 10 (CXCL10), MCP-1, and macrophage inflammatory protein 3 (MIP-3) in intestinal epithelial cells. These effects then allow a dramatic reduction in infiltrating inflammatory cells and lymphocytes in colitis [96]. However, whether fenofibrate can be applied to clinical treatment needs further exploration. In summary, fenofibrate protects against intestinal inflammation through the suppression of lymphocytes in the intestine (Table 3).

**Table 3:** Anti-inflammatory targets and mechanisms of fenofibrate action in digestive system diseases

**Diseases**	**Cells/Models**	**Targets and mechanisms**	**Implications**	**References**
NASH	Mouse	SREBP and LXRs and their target genes*↑*	Inhibit the accumulation of cholesterol in the liver	[26, 84]
	Mouse	PPAR-α↑	Reduce lipid accumulation and the secretion of modulating inflammatory chemokines	[86], [87]
	Mouse	NF-κB*↓*	Have anti-inflammatory effects	[9]
Cholestatic liver diseases	Human	PPAR-α↑	Affect bile acids production, make the components of bile acids healthier	[88]
	Rat	PPAR-α↑	Reduce serum cytokines, improve hepatic inflammation	[89]
	Mouse	PPAR-α↑-Cpt1b*↑*, Cpt2*↑*, Mcad*↑*-β-FAO*↑*	Reduce inflammation	[90]
Liver ischemia/reperfusion injury	Rat	TNF-α↓	Reduce oxidative stress, necrosis, and apoptosis	[91]
Hepatocellular adenomas	Human	IL-6/JAK/STAT pathway*↓*	Reduce inflammation	[92]
HCV-induced steatosis	Huh7 cells, Huh.8 cells	ER stress and inflammatory proteins*↓*- pAMPK and AdipoR2*↑*;	Reduce inflammation	[93]
Acute-phase response	Mouse	PPAR-α↑-IL-1*↓*- IL-6*↓*-ARP genes*↓*	Reduce systemic inflammation in the systemic APR	[32]
	Mouse	PPAR-α↑-IL-6 signaling pathway*↓*	Reduce systemic inflammation in the systemic APR	[94]
Intestinal inflammation	Mouse	Differentiation of Th17*↓*	Reduce inflammation	[95]
	Mouse, HT-29 Colorectal Cancer Cells	Th1*↓*,Th17*↓*- IFN-γ and IL-17*↓*; CXCL10*↓*, CCL2*↓*, CCL20*↓*	Reduce inflammatory cell infiltration and lymphocytes in colitis	[96]

NASH: Non-alcoholic steatohepatitis; SREBP: Sterol-regulatory element binding protein; LXR: Liver X receptor; PPAR-α: Peroxisome proliferator-activated receptor-α; NF-κB: Nuclear factor κ-B; Cpt1b: Carnitine palmitoyltransferase 1b; TNF-α: Tumor necrosis factor-α; IL: Interleukin; HCV: Hepatitis C virus; AdipoR2: Adiponectin receptor 2; IFN-γ: Interferon-γ; CXCL10: C-X-C motif chemokine ligand 10; APR: Acute phase response.

### Urinary system diseases

The anti-inflammatory effects of fenofibrate in urinary system diseases have been evaluated in studies focusing on kidney diseases, including diabetic nephropathy (DN), hypertensive renal injury, age-related kidney diseases, renal I/R injury, and lipotoxicity-induced renal injury.

DN, a most serious complication of diabetes, involves inflammatory processes. Researchers in the field showed that fenofibrate reduces inflammatory responses in DN by the inhibition of the NF-kB and transforming growth factor-β1 (TGF-β1)/SMAD family member 3 (Smad3)-signaling pathways independent of PPAR-α [97, 98]. Fenofibrate also exhibits protective effects against DN by upregulating the expression of (FGF21) which then induces the activation of the Akt2/GSK-3β/Fyn/Nrf2 and the AMPK pathways, both of which prevent renal inflammation [99, 100]. PPAR-α activation induced by fenofibrate is known to inhibit the inflammatory responses in DN by decreasing the expression of IL-18 [101] and the level of leukocytes adhering to mesangial cells [102].

In hypertensive renal injury, fenofibrate induces PPAR-α activation to reduce renal inflammatory responses in salt-loaded spontaneously hypertensive stroke-prone rats by protecting mitochondrial function [103]. In addition, fenofibrate can reduce oxidative stress and phosphorylation of MAPK to inhibit inflammation in hypertensive renal injury [104].

The kidney is sensitive to aging-related inflammation. Fenofibrate activates PPAR-α to evoke activated AMPK-SIRT1 signaling, thus attenuating age-related renal inflammation [105].

In renal I/R injury, fenofibrate plays a protective role in reducing inflammation via PPAR-α activation [106] that upregulates PI3K/Akt signaling and reduces the secretion of proinflammatory cytokines induced by IR injury [107].

Renal lipotoxicity occurs when the kidneys undergo excessive lipid accumulation. Fenofibrate inhibits this pathogenic mechanism in rats by upregulating the PPAR-α–FoxO3a–PGC-1α signal-transduction pathway [108, 109]. In addition, fenofibrate counteracts renal inflammation induced by lipotoxicity in HFD-fed mice by activating AMPK and autophagy, apparently independent of PPAR-α [110]. At present, the potential of fenofibrate for treating nephritic inflammation has not been clinically assessed. In summary, fenofibrate can significantly be used to diminish inflammation associated with nephropathy (Table 4).

**Table 4:** Anti-inflammatory targets and mechanisms of fenofibrate action in urinary system diseases

**Diseases**	**Cells/Models**	**Targets and mechanisms**	**Implications**	**References**
Diabetic nephropathy	Rat	NF-κB pathway*↓*, TGF-β1/Smad3 signaling pathways*↓*	Reduce inflammation	[97, 98]
	Rat	FGF21*↑*-Akt2/GSK-3β/Fyn/Nrf2 pathway*↑*, AMPK pathway*↑*	Prevent renal inflammation	[99, 100]
	Rat	PPAR-α↑-IL-18*↓*	Inhibit inflammatory responses	[101]
	Mouse	PPAR-α↑-leukocytes adherent to mesangial cells*↓*	Inhibit inflammatory responses	[102]
Hypertensive renal injury	Rat	PPAR-α↑	Reduce renal inflammatory responses	[103]
	Rat	Mitochondrial function*↑*	Reduce renal inflammatory responses	[33]
	Rat	Oxidative stress*↓*, phosphorylation of MAPK*↓*	Inhibit inflammation	[104]
Age-related renal injury	Mouse	PPAR-α↑, AMPK-SIRT1 signaling*↑*	Attenuate inflammation	[105]
Renal ischemia/reperfusion injury	Mouse	PPAR-α↑	Reduce inflammation	[106]
	Mouse	PPAR-α↑-PI3K/Akt signaling*↑*	Reduce the secretion of proinflammatory cytokines	[107]
Lipotoxicity-induced renal injury	Rat	PPAR-α↑	Reduce lipid accumulation	[108]
	Rat	PPAR-α–FoxO3a–PGC-1α pathway*↑*	Reduce lipid accumulation	[109]
	Mouse	AMPK*↑*-autophagy, FAO enzymes, and antioxidants*↑*	Reduce renal inflammation	[110]

NF-κB: Nuclear factor κ-B; TGF-β1: Transforming growth factor-β1; Smad3: SMAD family member 3; FGF21: Fibroblast growth factor 21; Nrf2: Nuclear factor erythroid-2-related factor 2; AMPK: Adenosine mitogen-activated protein kinase; PPAR-α: Peroxisome proliferator-activated receptor-α; IL: Interleukin; SIRT1: Sirtuin 1; MAPK: Mitogen-activated protein kinase; PI3K: Phosphoinositide 3-kinase; Akt: Protein kinase B; FoxO3a: Forkhead transcription factor O subfamily member 3a; PGC-1α: Peroxisome proliferator-activated receptor-γ coactivator -1α; FAO: Fatty acid oxidation.

### Immune system diseases

Fenofibrate exerts direct anti-inflammatory effects in immune diseases, and most of these effects are associated with PPAR-α [111].

In HIV-infected patients with hypertriglyceridemia, fenofibrate regulates chemokine gene expression in circulating leukocytes, thereby reducing the expression of C-C motif chemokine receptor 2 (CCR2) and C-X3-C motif chemokine ligand 1 (CX3CL1) to mitigate inflammatory responses [112]. This indicates that fenofibrate can suppress systemic inflammation in HIV-infected patients.

Rheumatoid arthritis manifests as acute or chronic connective tissue inflammation. Fenofibrate counters the inflammation in rheumatoid arthritis to improve its clinical symptoms [113–115]. PPAR-α is downregulated in inflammatory responses in arthritis; however, fenofibrate increases PPAR-α expression to inhibit arthritic inflammation [116].

Fenofibrate also reduces Müller cell proliferation and inflammatory cytokines secretion by Müller cells in rats with experimental autoimmune uveoretinitis. This effect is potentially associated with PPAR-α receptor stimulation [117]. In addition, in rats with experimental autoimmune myocarditis, fenofibrate restores Treg/Th17 balance by activating PPAR-α and inhibiting the NF-κB pathway [34]. The translation of fenofibrate into clinical use remains to be explored. In brief, fenofibrate principally counteracts inflammation in autoimmune diseases via PPAR-α activation (Table 5).

**Table 5:** Anti-inflammatory targets and mechanisms of fenofibrate action in immune system diseases

**Diseases**	**Cells/Models**	**Targets and mechanisms**	**Implications**	**References**
HIV-infected patients with hypertriglyceridemia	Human	CCR2 and CX3CL1*↓*	Attenuate inflammatory responses	[112]
Rheumatoid arthritis	Human	IL-6*↓*, CRP*↓*	Reduce arthritic inflammation	[115]
	Rat	PPAR-α↑	Reduce arthritic inflammation	[116]
Experimental autoimmune disease	Rat	PPAR-α↑-IL-6, IL-17 and VEGFs*↓*, Müller cell*↓*	Reduce inflammatory responses	[117]
	Rat	PPAR-α↑- NF-κB*↓*-Treg/Th17*↑*	Reduce inflammatory responses	[34]

CCR2: C-C motif chemokine receptor 2; CX3CL1: C-X3-C motif chemokine ligand 1; IL: Interleukin; PPAR-α: Peroxisome proliferator-activated receptor-α; NF-κB: Nuclear factor kappa-B; Th17: T helper cells 17; CRP: C-reactive protein; VEGF: Vascular endothelial growth factor.

### Nervous system diseases

In neurodegenerative diseases, fenofibrate decreases the expression of the two neuro-inflammatory genes—inducible NO synthase (iNOS) and COX-2, activating PPARs to exert neuroprotective effects [118]. The activation of PPAR-α induced by fenofibrate also improves peroxisomal function and reduces NO-induced neuroinflammation [119].

Traumatic brain injury (TBI) causes neuroinflammatory responses. The activation of PPAR-α by fenofibrate may counteract the deleterious inflammatory responses after TBI [120]. The expression of iNOS, COX-2, and MMP-9 is upregulated in TBI, but then reversed by fenofibrate; but whether these effects are associated with the activation of PPAR-α requires further examination [121].

Ischemic stroke can lead to neuroinflammation and cerebral injury. Chronic use of fenofibrate activates PPAR-α to diminish the expression of ICAM-1 and VCAM-1 [122]. Acute fenofibrate treatment also counters inflammation by reducing the infiltration of polymorphonuclear leukocytes (PMNs) and microglial activation after ischemic stroke. ICAM-1 is downregulated after fenofibrate intervention [123]. Fenofibrate improves stroke outcomes and resolves neuroinflammation more effectively in male than in female mice, which may be due to the lower PPAR-α expression in cells and tissues of females [124]. In addition, in rats with global cerebral I/R, fenofibrate suppresses p65 NF-κB and p38 MAPK activities, resulting in an anti-inflammatory effect [125].

Fenofibrate attenuates the neuroinflammation induced by intracerebral LPS injection and the expression of inflammation-related cytokines. The activities of macrophages and leukocytes are also weakened by fenofibrate through PPAR-α activation [126].

Multiple sclerosis is a chronic inflammatory demyelinating disease of the central nervous system. Astrocytes and microglia play crucial roles in the inflammatory responses typical of multiple sclerosis. Fenofibrate inhibits the production of NO induced by LPS, as well as the NF-κB DNA-binding activity and the secretion of the proinflammatory cytokines TNF-α, IL-1β, and IL-6 by astrocytes [127]. Moreover, NF-κB activity is suppressed by fenofibrate by activating SIRT1, but not PPAR-α [128]. Similar effects are also observed in microglia. Further, the inhibition on MCP-1 secretion after fenofibrate intervention impedes the migration of peripheral immune cells into the central nervous system [129].

A high-glucose environment favors the development of sciatic nerve inflammation, which may be attenuated by fenofibrate by triggering the PPAR-α-AMPK-PGC-1α-eNOS pathway, followed by the activation of the PI3K-Akt-eNOS-signaling pathway [130].

In a PPAR-α-dependent manner, fenofibrate also leads to the underproduction of IL-1β and TNF-α in the brains of patients with Huntington’s disease [131]. In summary, fenofibrate can effectively reduce neuroinflammation, which endows it with clinical therapeutic potential (Table 6).

**Table 6:** Anti-inflammatory targets and mechanisms of fenofibrate action in nervous system diseases

**Diseases**	**Cells/Models**	**Targets and mechanisms**	**Implications**	**References**
Neurodegenerative diseases	Mouse	PPAR-α↑- iNOS*↓*, COX-2*↓*	Generate neuroprotective effects	[118]
	Cortical neurons	PPAR-α↑- peroxisomal activity*↑*	Reduce neuroinflammation	[119]
Traumatic brain injury	Rat	PPAR-α↑	Reduce inflammatory responses	[120]
	Rat	iNOS, COX-2 and matrix metalloproteinase-9 (MMP-9) *↓*	Reduce inflammatory responses	[121]
Ischemic strokes	Mouse	PPAR-α↑- ICAM-1*↓*,VCAM-1*↓*	Have anti-inflammatory effects	[122]
	Rat, mouse	PMNs infiltration and microglial activation*↓*, ICAM-1*↓*	Reduce inflammatory responses	[123]
Global cerebral ischemia/reperfusion	Rat	P65 NF-κB*↓*, p38 MAPK*↓*	Have anti-inflammatory effects	[125]
Neuroinflammation induce by intracerebral LPS injection	Mouse	PPAR-α↑	Attenuate neuroinflammation	[126]
Multiple sclerosis	Murine astrocytes	NO*↓*,NF-κB DNA binding activity*↓*	Reduce inflammatory responses	[127]
	Murine microglia	SIRT1*↑*- NF-κB*↓*	Reduce inflammatory responses	[128]
	Murine microglia	MCP-1*↓*	Impede the migration of peripheral immune cells into the central nervous system	[129]
Diabetic peripheral neuropathy	Mouse, HUVECs, HSCs	PPAR-α-AMPK-PGC-1a-eNOS pathway*↑*-PI3K-Akt-eNOS signaling*↑*	Reduce inflammatory responses	[130]
Huntington’s disease	Rat	PPAR-α↑- IL-1β↓, TNF-α↓	Reduce inflammatory responses	[131]

PPAR-α: Peroxisome proliferator-activated receptor α; iNOS: Inducible nitric oxide synthase; ICAM-1: Intercellular cell adhesion molecule 1; PMN: Polymorphonuclear leukocyte; NF-κB: Nuclear factor κ-B; NO: Nitric oxide; SIRT1: Sirtuin 1; HUVEC: Human umbilical vein endothelial cell; AMPK: Adenosine mitogen-activated protein kinase; COX-2: Cyclooxygenase-2; VCAM - 1: Vascular cell adhesion protein 1; MAPK: Mitogen-activated protein kinase; MPC -1: Monocyte chemoattractant protein 1; PGC: Primordial germ cell; eNOS: Endothelial nitric oxide synthase; PI3K: Phosphoinositide 3-kinase; IL-1: Interleukin 1; TNF-α: Tumor necrosis factor α; LPS: Lipopolysaccharide; CNS: Central nervous system; HCS: Human chorionic somatomammotropin.

### Respiratory system diseases

Fenofibrate inhibits inflammation in the respiratory system by upregulating PPAR-α [132]. By means of PPAR-α, fenofibrate downregulates cellular infiltration and chemoattractant production, thus enhancing MMP activity triggered by LPS in the mouse lung [133]. Moreover, fenofibrate lowers the level of IL-1β to reduce neutrophilic inflammation [134].

In treating bronchial asthma, fenofibrate is more effective than dexamethasone, whose effect is partially mediated by suppression of Th17-IL-23/IL-17 axis. This superiority is achieved by modulation of both Th2- and Th17-cell-derived cytokines [135]. Fenofibrate dose-dependently blocks inflammatory cell infiltration in asthmatic airways. Fenofibrate also dramatically reduces inflammatory responses, including infiltration of eosinophils, neutrophils, lymphocytes, and macrophages, and increases the release of IL-4, IL-5, TNF-α, MIP-2, and MCP-1 [136]. These beneficial effects may bring a possibility of using fenofibrate to design anti-asthma drug. In conclusion, fenofibrate can significantly improve the inflammation of the respiratory system (Table 7).

**Table 7:** Anti-inflammatory targets and mechanisms of fenofibrate action in respiratory system diseases

**Diseases**	**Cells/Models**	**Targets and mechanisms**	**Implications**	**References**
Airway inflammation	Mouse	PPAR-α↑	Inhibit inflammation	[132, 133]
	Human small airway epithelial cells	IL-1β production*↓*	Reduce neutrophilic inflammation	[134]
Asthma	Rat	TH17-IL-23/IL-17 axis*↓*	Have anti-inflammatory effects	[135]
	Mouse	Infiltration of eosinophils*↓*, neutrophils*↓*, lymphocytes*↓*, macrophages, IL-4, IL-5, TNF-α, MIP-2, and MCP-1*↓*	Reduce inflammatory responses	[136]

PPAR-α: Peroxisome proliferator-activated receptor α; IL: Interleukin; TNF-α: Tumor necrosis factor-α; MCP-1: Monocyte chemoattractant protein 1; MIP-2: Macrophage inflammatory protein 2.

**Table 8:** Anti-inflammatory targets and mechanisms of fenofibrate action in bacterial sepsis

**Diseases**	**Cells/Models**	**Targets and mechanisms**	**Implications**	**References**
Bacterial sepsis	Mouse	ERK phosphorylation*↓*- GRK2*↓*- CXCR2*↑*	Promote neutrophil accumulation at infection sites, quick eliminate pathogens	[137]
	Mouse, bone marrow-derived macrophages	LKB1*↑*-pAMPK*↑*-SHP*↑*- NF-κB*↓*, UCP2*↑*	Reduce inflammatory responses	[138]
	Rabbit	Monocyte tissue factor*↓*	Block endothelial inflammation	[139]
	Mouse	TNF*↓*	Decrease endotoxin action	[140]

ERK: Extracellular-signal-regulated kinase; GRK2: G protein-coupled receptor kinases 2; CXCR2: C-X-C motif chemokine receptor 2; LKB1: Liver kinase B1; SHP: Small heterodimer partner; NF-κB: Nuclear factor κ-B; UCP2: Uncoupling protein 2; TNF: Tumor necrosis factor.

### Bacterial sepsis

Bacterial sepsis is a systemic inflammation induced by infection. Fenofibrate reduces this inflammation by enhancing neutrophil chemotaxis. Independent of PPAR-α, fenofibrate inhibits the downregulation of C-X-C motif chemokine receptor 2 (CXCR2) by blocking extracellular-signal-regulated kinase (ERK) phosphorylation and subsequent expression of G protein-coupled receptor kinases 2 (GRK2) and promotes neutrophil accumulation at infection sites and quick elimination of pathogens [137]. Moreover, fenofibrate activates the liver kinase B1 (LKB1)/AMPK pathway and increases small heterodimer partner (SHP) expression. SHP then downregulates NF-kB and upregulates mitochondrial uncoupling protein 2 (UCP2) to reduce ROS production, providing the inhibitory effects on inflammatory responses [138]. Fenofibrate also represses monocyte tissue factor expression to block endothelial inflammation in endotoxin-induced shock by the activation of PPAR-α [139]. In addition, by activating PPAR-α, fenofibrate also blunted the upregulation of TNF, which is proved to be a proximal mediator of endotoxin action [140].

Although fenofibrate effectively reduces the systematic inflammation in bacterial sepsis, this effect is limited to inflammation induced by pathogens; in contrast, neutrophil-dependent sterile inflammation does not subside after fenofibrate treatment [137] (Table 8).

**Figure 1.**
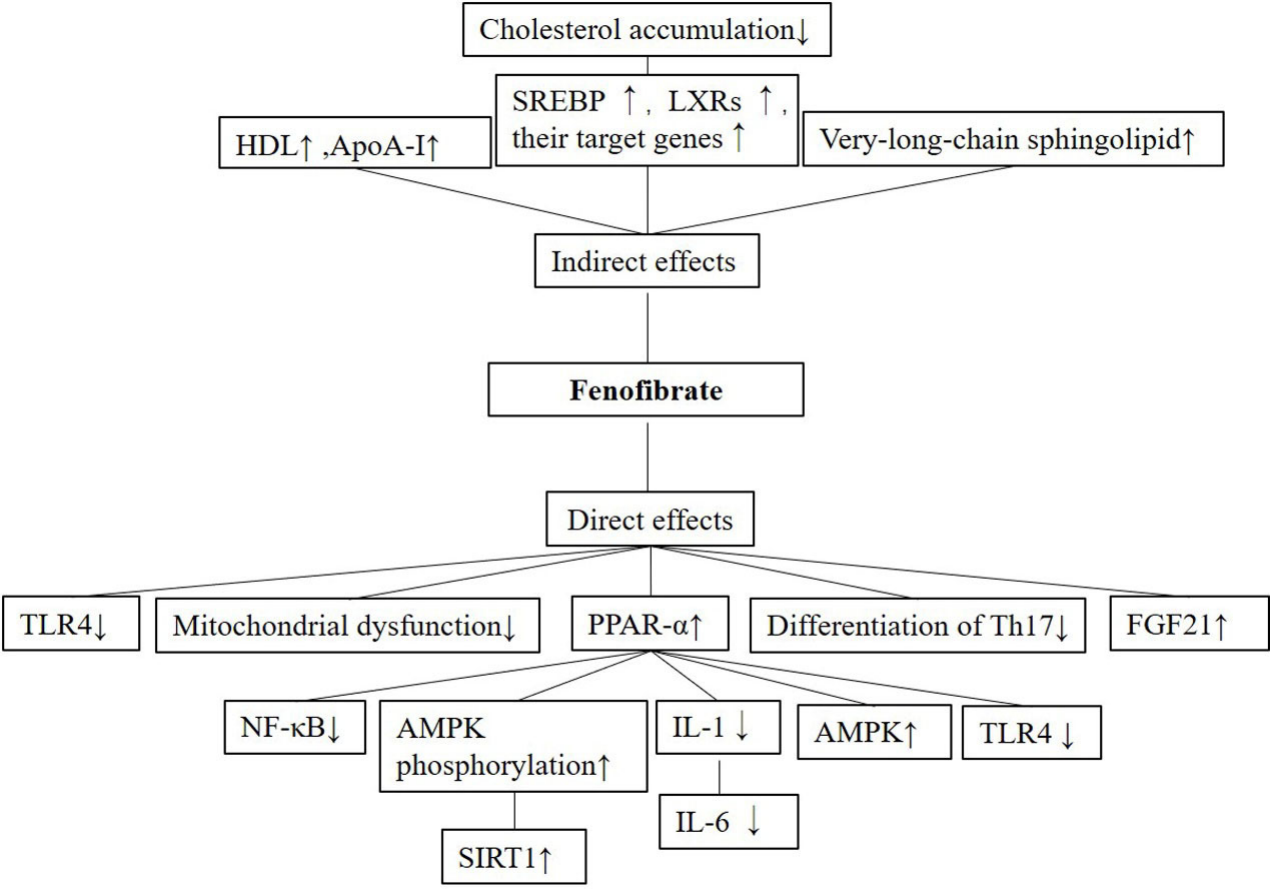
**A schematic figure of the mechanisms governing the anti-inflammatory effects (direct or indirect) of fenofibrate in various diseases.** HDL: High-density lipoprotein; ApoA-I: Apolipoprotein A-I; SREBP: Sterol-regulatory element binding protein; LXR: Liver X receptors; TLR4: Toll-like receptor 4; PPAR-α: Peroxisome proliferator-activated receptor α; FGF21: Fibroblast growth factor 21; NF-κB: Nuclear factor κ-B; AMPK: Adenosine mitogen-activated protein kinase; SIRT1: Sirtuin 1.

## Discussion

Fenofibrate, a PPAR-α agonist, has been primarily used to control diseases related to metabolism, and its anti-inflammatory properties have been observed in various diseases. Its action against inflammation is chiefly achieved via the activation of PPAR-α and the regulation of associated inflammatory pathways. Fenofibrate also functions independently of PPAR-α. Fenofibrate suppresses inflammatory responses by modulating cytokines secretion, cellular infiltration, cell death, and organ dysfunction. The advantage of fenofibrate treatment lies in simultaneous regulation of various metabolic disorders and few side effects. The direct or indirect anti-inflammatory effects of fenofibrate have been observed in atherosclerosis, diabetic retinopathy, NASH, etc. However, most of these studies are at the animal or cell level. In clinical practice, many patients with abnormal lipid metabolism also suffer from these diseases. Therefore, whether fenofibrate can be used to treat these diseases requires human or clinical trials.

Although fenofibrate shows extensive anti-inflammatory effects in many diseases, it is not always protective against inflammation. In some studies, fenofibrate exerts no effects [141], or even exacerbates the inflammatory response [142–144]. For example, fenofibrate, at a dose commonly used for lipid abnormalities and heart disease, does not suppress inflammation in response to low-dose endotoxin in healthy humans. These results suggest that the anti-inflammatory effects of fenofibrate in systemic inflammation are limited [145] compared to those in local inflammation.

## Conclusion

Here we summarized the anti-inflammatory effects and mechanisms of fenofibrate in various systemic diseases (Figure 1). As a blood lipid-lowering drug used clinically, fenofibrate exerts an anti-inflammatory effect, which is secondary to the decrease of blood lipid, to reduce the inflammation induced by hyperlipidemia. As a PPAR-α agonist, fenofibrate plays most of its roles through activating PPAR-α and inhibiting downstream inflammatory signaling pathways. In addition to these indirect effects, fenofibrate can also directly regulate inflammation-related signaling pathways. Fenofibrate is not the first choice for many diseases we discussed in this review. Moreover, many studies on its efficacy are still at the animal or cellular level. Thus, the therapeutic potential of fenofibrate should be validated with more basic research and clinical trials.
